# A *Pax3* lineage gives rise to transient haematopoietic progenitors

**DOI:** 10.1242/dev.202924

**Published:** 2024-12-04

**Authors:** Giovanni Canu, Rosamaria Correra, Guillermo Diez-Pinel, Raphaël F. P. Castellan, Laura Denti, Alessandro Fantin, Christiana Ruhrberg

**Affiliations:** ^1^ UCL Institute of Ophthalmology, University College London, London EC1V 9EL, UK; ^2^ Department of Biosciences, University of Milan, 20133 Milan, Italy

**Keywords:** Pax3, Foetal liver, Hematopoietic development, Macrophage origin, Paraxial mesoderm, Vascular endothelial cell, Mouse

## Abstract

During embryonic development, muscle tissues, skin, and a subset of vascular endothelial cells arise from *Pax3*-expressing embryonic progenitors defined as paraxial mesoderm. By contrast, haemogenic potential is well established for extra-embryonic mesoderm and intra-embryonic lateral plate mesoderm, which do not express *Pax3*. To date, it is not known whether the haematopoietic system also contains *Pax3* lineage cells. Here, we show that the mouse foetal liver and foetal circulation contain a transient population of *Pax3* lineage cells with hallmarks of haematopoietic progenitors and the potential to generate both myeloid and erythroid cells. We propose that *Pax3* lineage haematopoietic cells should be investigated to better understand normal haematopoietic development from different mesodermal derivatives. Further, genetic alterations of *Pax3* lineage haematopoietic cells should be investigated for their potential to cause haematopoietic malignancies.

## Teaser

**Summary:** Pax3-expressing cells generate neural crest and paraxial mesoderm. Here, we show that they also produce transient haematopoietic progenitors capable of generating embryonic myeloid and erythroid cells.

## INTRODUCTION

Deducing cell lineages in developmental biology research is commonly achieved with genetic lineage tracing in the mouse via Cre/LoxP-mediated DNA recombination (Li et al., 2018), whereby Cre recombinase expressed from a cell type-specific mouse promoter irreversibly excises a stop codon upstream of a fluorescent reporter, such as *Rosa^Egfp^*, *Rosa^Yfp^* or *Rosa^tdTom^* (Madisen et al., 2010). Using this method with the *Pax3^Cre^* knock-in allele (Lang et al., 2005), it has been shown that muscle cells and a subset of endothelial cells arise from *Pax3-*expressing paraxial mesoderm, whereas melanocytes and a subset of neurons arise from *Pax3*-expressing neural crest (Conway et al., 1997; Wu et al., 2008; Mayeuf-Louchart et al., 2014; Stone and Stainier, 2019; Tani et al., 2020). Recently, bulk RNA-sequencing (RNA-seq) of *Pax3^Cre^* lineage-traced embryonic mouse limbs identified unexplained transcripts for ‘immune system-related genes’ alongside the expected musculoskeletal and neuronal markers typical of paraxial mesoderm and neural crest derivatives (Singh et al., 2018).

During embryonic development, haematopoiesis occurs in temporally and spatially overlapping waves that originate from well-defined tissues (Canu and Ruhrberg, 2021). Extra-embryonic mesoderm gives rise to yolk sac haemogenic endothelium that transitions into pro-definitive haematopoietic progenitors, including erythro-myeloid progenitors (EMPs) and lympho-myeloid progenitors (LMPs) (Zambidis et al., 2006; Canu and Ruhrberg, 2021; Biben et al., 2023). By contrast, intra-embryonic lateral plate mesoderm produces haemogenic endothelium in the dorsal aorta that transitions into definitive haematopoietic stem cells (HSCs) (Wasteson et al., 2008; Seco et al., 2020; Canu and Ruhrberg, 2021). Neither type of mesoderm with haematopoietic potential is known to express *Pax3*, which instead is considered to be selectively expressed in paraxial mesoderm and neural crest (Conway et al., 1997; Wu et al., 2008; Mayeuf-Louchart et al., 2014; Stone and Stainier, 2019; Tani et al., 2020).

Here, we show that the mouse foetal liver and foetal circulation contain a transient population of *Pax3* lineage cells with hallmarks of haematopoietic progenitors and the potential to generate both myeloid and erythroid cells. Our findings raise the possibility that haemogenic potential, observed for extra-embryonic mesoderm and intra-embryonic lateral plate mesoderm, may also extend to paraxial mesoderm.

## RESULTS AND DISCUSSION

**Fig. 1.**
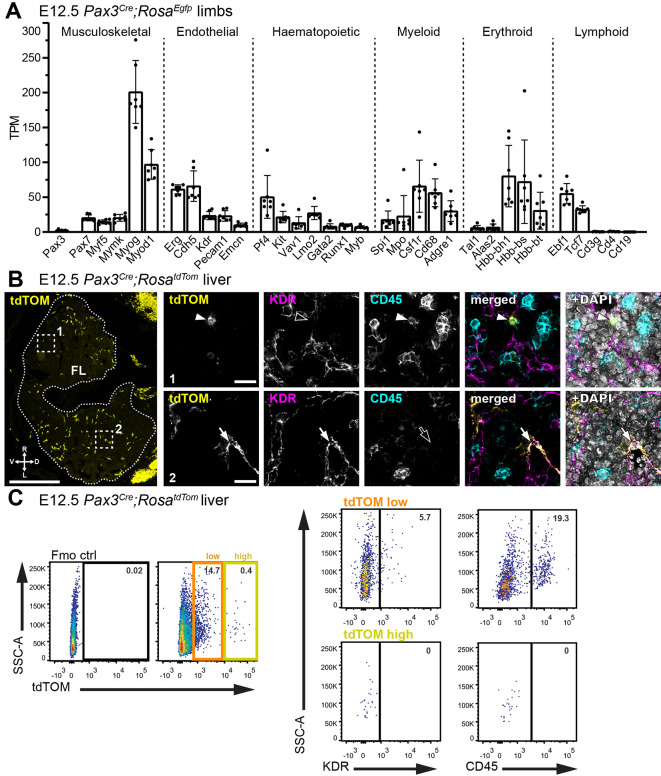
***Pax3* lineage endothelial and haematopoietic cells in the foetal liver and blood.** (A) Bulk RNA-seq analysis of EGFP^+^ cells from E12.5 *Pax3^Cre^;Rosa^Egfp^* mouse limbs (ENA project PRJNA422253) for transcripts from the indicated genes. Data are shown as mean±s.d.; *n*=7 embryos. (B) Representative immunofluorescence staining with the indicated markers of a E12.5 *Pax3^Cre^;Rosa^tdTom^* embryo section at the level of the foetal liver (FL, dotted outline); *n*=3 embryos. The dorsal (D), ventral (V), right (R) and left (L) side of the embryo are indicated. The dashed squares indicate two areas shown at higher magnification in the adjacent panels to visualise tdTOM^+^ CD45^+^ haematopoietic cells (arrowheads in panel 1) and tdTOM^+^ KDR^+^ endothelial cells (arrows in panel 2). (C) Representative dot plots of flow cytometry analysis from E12.5 *Pax3^Cre^;Rosa^tdTom^* liver, showing the proportion of endothelial and haematopoietic cells in the tdTOM low and tdTOM high fractions; *n*=5 embryos for KDR, *n*=10 embryos for CD45. Scale bars: 500 µm (B); 20 µm (B, insets).

Re-analysis of bulk RNA-seq data (Singh et al., 2018) from FACS-isolated embryonic day (E) 12.5 mouse limbs genetically lineage traced with *Pax3^Cre^;Rosa^Egfp^* detected the expected transcripts for the paraxial mesoderm-derived musculoskeletal (e.g. *Myf5*, *Myog*) and endothelial (e.g. *Cdh5*, *Kdr*) cell lineages, but also transcripts typical of haematopoietic progenitors (e.g. *Kit*, *Vav1*, *Runx1*), myeloid cells (e.g. *Csf1r*, *Cd68*) and erythroid cells (e.g. *Hbb-bh1*, *Hbb-bs*; Fig. 1A). Transcription factors required for lymphoid differentiation were also detected (e.g. *Ebf1*, *Tcf7*), but not markers of mature lymphocytes (e.g. *Cd3g*, *Cd4*, *Cd19*; Fig. 1A). The finding suggests that a *Pax3* lineage may give rise to a subset of haematopoietic cells.

**Fig. 2.**
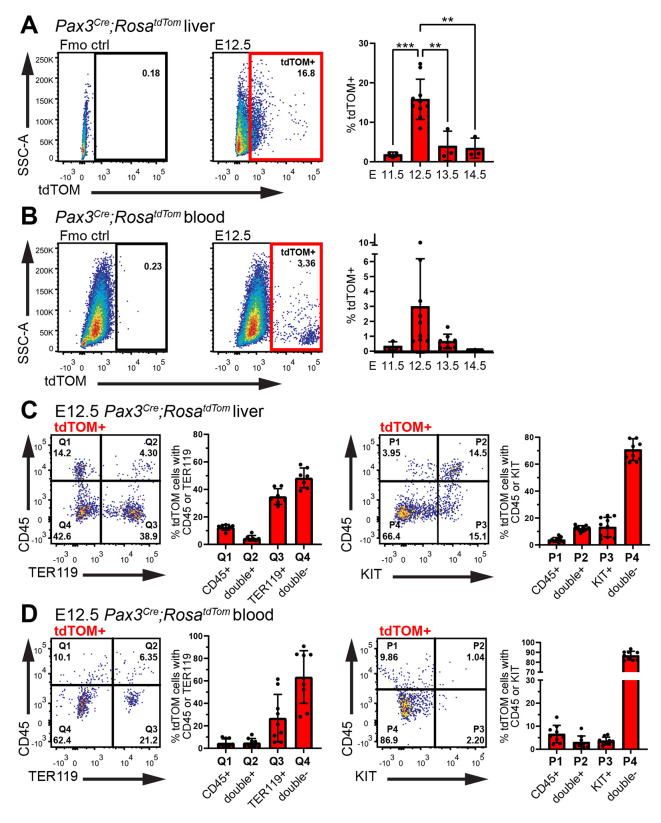
**Transience of *Pax3* lineage haematopoietic cells in the foetal liver and blood.** (A-D) Flow cytometry analysis of *Pax3^Cre^;Rosa^tdTom^* liver (A,C) and blood (B,D) including representative dot plots of E12.5 tdTOM^+^ cells with quantitative analysis of E11.5-E14.5 tdTOM^+^ cells (A,B), and quantitative analysis of E12.5 tdTOM^+^ CD45^+^ TER119^+^ KIT^+^ cells (C,D). Data are shown as mean±s.d.; each data point represents the value from the liver or blood of an individual embryo; liver: *n*=3 E11.5, *n*=10 E12.5, *n*=3 E13.5, *n*= 3 E14.5; blood: *n*=3 E11.5, *n*=9 E12.5, *n*=8 E13.5, *n*=3 E14.5. **P<0.01, ***P<0.001 (one-way ANOVA).

We next examined the *Pax3* lineage trace in the mouse foetal liver, which harbours haematopoietic progenitors from midgestation until birth to support foetal haematopoiesis (Canu and Ruhrberg, 2021). Immunofluorescence staining identified *Pax3* lineage KDR^+^ endothelial and CD45^+^ haematopoietic cells in E12.5 *Pax3^Cre^;Rosa^tdTom^* liver (Fig. 1B). Flow cytometry corroborated the presence of *Pax3* lineage KDR^+^ and CD45^+^ cells in E12.5 liver (Fig. 1C; 46.98±11.36% of KDR^+^ endothelial cells and 25.70±7.15% of CD45^+^ haematopoietic cells in the E12.5 liver were lineage-traced with *Pax3^Cre^*). A small fraction of cells (0.40±0.10%) in the foetal liver expressed higher levels of tdTOM, but these cells were negative for both KDR or CD45, excluding endothelial and haematopoietic identity (Fig. 1C). *Pax3* lineage cells were also identified in the circulation but were less abundant than in the liver (E12.5 liver: 15.83±5.09%; E12.5 blood: 2.99±3.20%; Fig. 2A,B). In both the liver and blood, the proportion of *Pax3* lineage cells peaked at E12.5 and then rapidly declined (Fig. 2A,B). We investigated the identity of *Pax3* lineage cells at E12.5 and determined that 16.89±3.46% of *Pax3* lineage cells in the liver and 9.64±4.88% in the blood were CD45^+^ haematopoietic cells (Fig. 2C,D). The *Pax3* lineage-traced populations at both sites also included TER119^+^ erythroid cells (E12.5 liver: 39.31±5.73%; E12.5 blood: 31.84±24.18%; Fig. 2C,D). Moreover, a subset of CD45^+^ *Pax3* lineage cells co-expressed the haematopoietic progenitor marker KIT (Fig. 2C,D). Consistent with haematopoietic progenitors being enriched in the foetal liver at midgestation (Canu and Ruhrberg, 2021), *Pax3* lineage CD45^+^KIT^+^ cells were more abundant in the liver than blood (E12.5 liver: 12.27±2.00%; E12.5 blood: 3.06±2.73%; Fig. 2C,D).

**Fig. 3.**
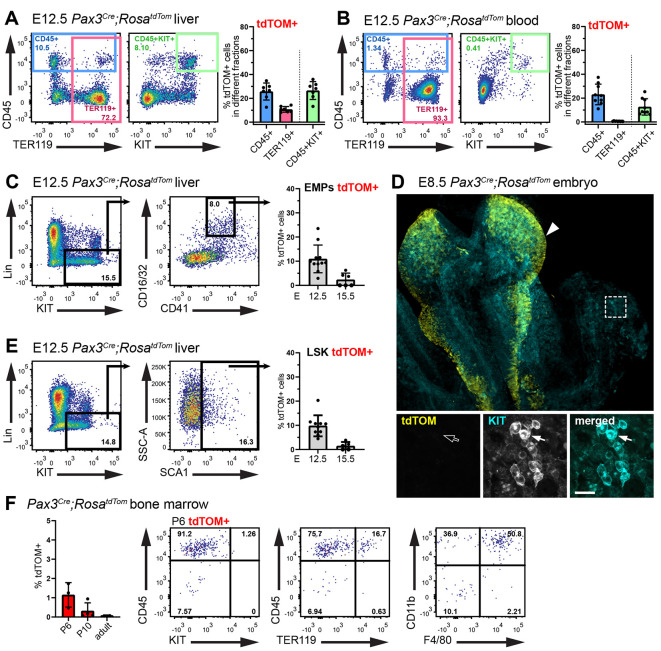
**The *Pax3* lineage contributes haematopoietic progenitors to the foetal liver and blood but not postnatal bone marrow.** (A,B) Flow cytometry analysis of E12.5 *Pax3^Cre^;Rosa^tdTom^* liver (A) and blood (B), including representative dot plots and quantitative analysis of the fractions of all CD45^+^, TER119^+^ and CD45^+^ KIT^+^ cells expressing tdTOM. Liver: *n*=7; blood: *n*=9. (C) Flow cytometry analysis of E12.5 *Pax3^Cre^;Rosa^tdTom^* liver, including representative dot plots and quantitative analysis of the fraction of EMPs expressing tdTOM. E12.5: *n*=11; E15.5: *n*=6. (D) Representative immunofluorescence staining with the indicated markers of an E8.5 *Pax3^Cre^;Rosa^tdTom^* embryo (dorsal view) and yolk sac; *n*=2 independent embryos. The dashed square indicates a yolk sac area displayed at higher magnification in the panels below, including separate channels for tdTOM and KIT in greyscale. Emerging KIT^+^ EMPs (arrow) are tdTOM^−^, whereas tdTOM^+^ cells are visible in the dorsal neural tube (arrowhead). (E) Flow cytometry analysis of E12.5 *Pax3^Cre^;Rosa^tdTom^* liver, including representative dot plots and quantitative analysis of the fraction of LSK cells expressing tdTOM. E12.5: *n*=10; E15.5: *n*=5. (F) Flow cytometry analysis of *Pax3^Cre^;Rosa^tdTom^* bone marrow, including quantitative analysis of P6, P10 and adult (10 months old) tdTOM^+^ cells, and representative dot plots of P6 tdTOM^+^ cells. P6: *n*=3, P10: *n*=4; adult: *n*=2. Data in A-C,E,F are shown as mean±s.d.; each data point represents the value from the blood or liver of an individual embryo or mouse. Scale bar: 20 µm.

Next, we quantified the contribution of *Pax3* lineage cells to the developing haematopoietic system at E12.5. In the liver, more than 25% of CD45^+^ cells overall and CD45^+^KIT^+^ progenitors were *Pax3* lineage-traced (CD45^+^: 25.70±7.15%; CD45^+^KIT^+^: 26.49±7.47%; Fig. 3A). The proportion of *Pax3* lineage-traced CD45^+^KIT^+^ cells in the blood was approximately half of that in the liver at E12.5 (12.63±6.89%, Fig. 3B), again consistent with haematopoietic progenitor enrichment in the midgestation foetal liver. *Pax3* lineage TER119^+^ erythroid cells were present in the E12.5 liver but rare in the blood (liver: 10.71±2.38%; blood: 0.52±0.27%; Fig. 3A,B). Considering that most TER119^+^ cells in the E12.5 circulation are primitive erythrocytes (Canu and Ruhrberg, 2021), foetal liver presence of *Pax3* lineage TER119^+^ cells, but their absence from the circulation, suggests that *Pax3* lineage haematopoietic cells are not derived from the so-called primitive haematopoietic wave. Instead, these findings suggest that *Pax3* lineage haematopoietic cells home to the foetal liver to differentiate into TER119^+^ erythrocytes, as described for erythroid cell production from pro-definitive EMPs at midgestation (Canu and Ruhrberg, 2021). Although *Pax3* lineage haematopoietic cells expressed markers typical of EMPs (Lin^−^KIT^+^CD16/32^+^CD41^low^) in the foetal liver (10.95±5.73%; Fig. 3C), these cells were not detected in the E8.5 yolk sac, where EMPs arise (Fig. 3D). These findings suggest that *Pax3* lineage cells are EMP-like progenitors generated from a haemogenic source inside the embryo, or that they express Pax3 only after leaving the yolk sac.

During foetal development, HSCs home to the foetal liver from around E12.5 onwards, before they seed the bone marrow to sustain adult haematopoiesis (Canu and Ruhrberg, 2021). *Pax3* lineage cells contributed to the LSK (Lin^−^SCA1^+^KIT^+^) haematopoietic stem/progenitor cell pool in the E12.5 foetal liver (9.81±4.36%; Fig. 3E). Therefore, we next investigated whether *Pax3* lineage haematopoietic progenitors also migrated to the bone marrow. Flow cytometry of postnatal day (P) 6 *Pax3^Cre^;Rosa^tdTom^* bone marrow detected rare *Pax3* lineage cells (1.15±0.64%) that were mostly CD45^+^CD11b^+^F4/80^+^ macrophages and CD45^+^CD11b^+^F4/80^−^ myeloid cells; by contrast, *Pax3* lineage KIT^+^CD45^+^ progenitors or TER119^+^CD45^−^ erythrocytes were not observed (Fig. 3F). Fewer *Pax3* lineage cells were detected in P10 than P6 bone marrow, and they were undetectable in adult bone marrow (Fig. 3F). Absent adult bone marrow colonisation is inconsistent with *Pax3* lineage haematopoietic progenitors being a subset of HSCs but consistent with a pro-definitive, EMP-like identity.

**Fig. 4.**
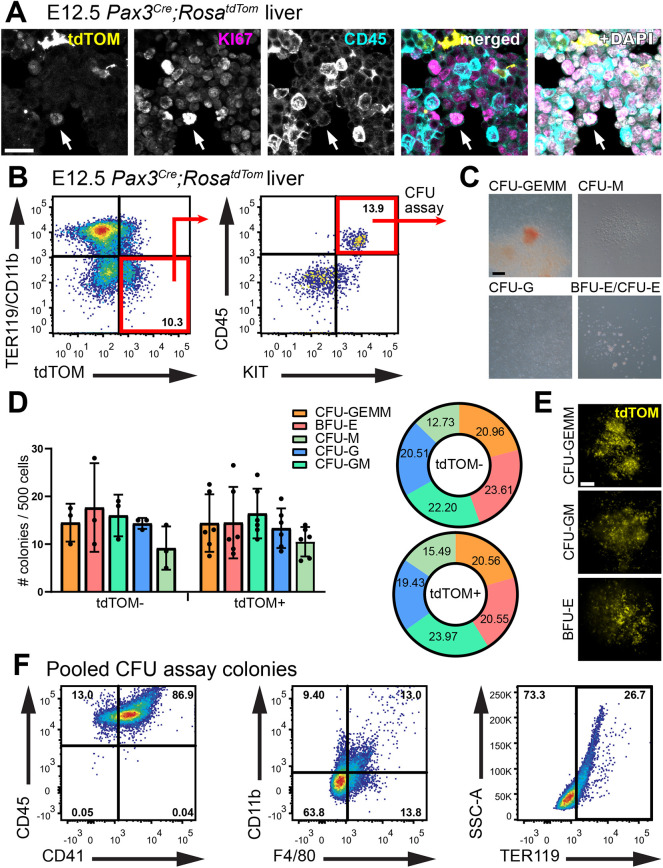
***Pax3* lineage cells have hallmarks of multipotent haematopoietic progenitors.** (A) Representative immunofluorescence staining with the indicated markers of a E12.5 *Pax3^Cre^;Rosa^tdTom^* embryo section at the level of the foetal liver; *n*=2 embryos. The arrow indicates a tdTOM^+^ CD45^+^ cell expressing the proliferation marker KI67. (B-F) *Pax3* lineage (tdTOM^+^) TER119^−^ CD11b^−^ KIT^+^ CD45^+^ haematopoietic progenitors were isolated from E12.5 *Pax3^Cre^;Rosa^tdTom^* liver for CFU assays. (B) Representative dot plots highlight sorted *Pax3* lineage cells (red squares). (C) Representative brightfield images of CFU assay colonies. (D) Quantitative analysis of colonies scored after 12 days for tdTOM^−^ and tdTOM^+^ progenitors; bar plot data are shown as mean±s.d.; each data point represents the number of colonies derived from a pooled litter of 5-7 embryos; tdTOM^−^: *n*=3 litters; tdTOM^+^: *n*=6 litters. Pie chart shows the frequency for each colony type as mean percentage of total colonies obtained. (E) Fluorescence image of representative CFU colonies. (F) Representative dot plots of flow cytometry analysis from pooled CFU colonies with the indicated markers to show the presence of differentiated blood cell types; *n*=3 CFU assays. CFU-GEMM, granulocyte-erythrocyte-macrophage-megakaryocyte; BFU-E, burst-forming unit erythrocyte; CFU-E, eyrthrocytes; CFU-GM, granulocyte-macrophage; CFU-G, granulocyte; CFU-M, macrophage. Scale bar: 20 µm (A); 200 µm (C); 300 µm (E).

Immunofluorescence staining of E12.5 liver showed that *Pax3* lineage CD45^+^ haematopoietic cells expressed KI67, a marker of proliferating cells (Fig. 4A), indicating that they could be isolated and placed into colony-forming unit (CFU) assays to determine their haematopoietic potential. Thus, we isolated E12.5 liver CD45^+^KIT^+^ cells lacking differentiated lineage markers for CFU assays, in which they gave rise to multilineage CFU-GEMM along with erythroid and myeloid colonies (Fig. 4B-D). The number and type of colonies produced was similar for tdTOM^−^ and tdTOM^+^ progenitors (Fig. 4D). Detecting uniform tdTOM expression in differentiated CFU colonies excluded the possibility that the haematopoietic potential of explanted progenitors was derived from contaminating tdTOM^−^ cells (Fig. 4E). Further, the *Pax3* lineage CFU colonies expressed blood lineage markers consistent with differentiated erythroid and myeloid cells (Fig. 4F). Finding that *Pax3* lineage haematopoietic progenitors have erythroid and myeloid differentiation potential, in the context of their transient presence in the foetal liver but absence from bone marrow, again agrees with the idea that these cells are more similar to pro-definitive EMPs than definitive HSCs. The residual presence of *Pax3* lineage macrophages in the neonatal bone marrow, in the absence of *Pax3* lineage haematopoietic progenitors, is reminiscent of EMP-derived macrophages, which persist as tissue-resident in adults in an organ-specific manner even though their progenitors are only transient (Canu and Ruhrberg, 2021). Nevertheless, future studies should investigate whether *Pax3* lineage macrophages also persist in adult organs.

As *Pax3* is an established marker of paraxial mesoderm and neural crest, both populations might be considered as potential sources of *Pax3* lineage haematopoietic progenitors. In particular, it is conceivable that these progenitors might arise from a subset of paraxial mesoderm-derived endothelial cells with haemogenic potential, in a process analogous to that described for extra-embryonic mesoderm-derived haemogenic endothelium in the yolk sac or lateral plate mesoderm-derived haemogenic endothelium in the dorsal aorta (Canu et al., 2020; Seco et al., 2020; Biben et al., 2023). Alternatively, *Pax3* may be expressed in a distinct and hitherto unrecognised source of progenitors with haematopoietic potential that may or may not pass through an endothelial intermediate, in which case *Pax3*-mediated lineage tracing would be insufficient to attribute cellular origins to paraxial mesoderm.

We have not investigated whether *Pax3* lineage haematopoietic progenitors may be involved in haematopoietic malignancies. Nevertheless, it is conceivable that genetic alterations that cause leukemic transformation in haematopoietic stem and progenitor cells might also affect *Pax3* lineage haematopoietic progenitors. Furthermore, the *Pax3* lineage origin of these transient progenitors raises the possibility that they may also be affected by genetic alterations that are not commonly taken into consideration for haematological studies. In this context, we note that a t(2;13)(q35;q14) genetic translocation produces a tumorigenic PAX3-FOXO1 fusion protein in patients with paediatric alveolar rhabdomyosarcoma (aRMS) (Douglass et al., 1987; Skapek et al., 2019). This mutation increases the proliferation of *Pax3*-expressing cells while inhibiting their terminal differentiation (Milewski et al., 2021) and also promotes the trans-differentiation of endothelial cells towards a myogenic fate (Searcy et al., 2023). Thus, it should be examined whether PAX3-FOXO1 could also induce myogenic trans-differentiation in haematopoietic cells, or whether it might alter haematopoietic development via an endothelial-derived *Pax3* lineage haematopoietic progenitor. These are interesting considerations, because some aRMS patients with PAX3-FOXO1 fusion protein have a bone marrow phenotype resembling acute leukaemia (Sandberg et al., 2001), which is currently thought to result from metastatic aRMS infiltrating the bone marrow, even in cases with no identifiable primary tumour (Karagiannis et al., 2015). Existing mouse models (Keller et al., 2004; Nishijo et al., 2009) could be used to determine whether such translocation can cause primary haematopoietic malignancies.

### Conclusion

Here, we have identified *Pax3* lineage cells with hallmarks of transient embryonic haematopoietic progenitors. Our results should open new lines of investigations to determine the origins and role of *Pax3* lineage progenitors during normal haematopoietic development, and their relevance for haematological malignancies.

## MATERIALS AND METHODS

### Transcriptomic studies

Raw bulk RNA-seq reads from *Pax3^Cre^;Rosa^Egfp^* mouse embryo limbs (PRJNA422253) (Singh et al., 2018) were downloaded from the European Nucleotide Archive, aligned to GRCm39 and annotated using Mus_musculus.GRCm39.110.gtf at http://www.ensembl.org/info/data/ftp/index.html. Transcripts per million (TPM) values were plotted in Prism 9 (GraphPad).

### Animal procedures and tissue staining

Animal procedures were performed with Animal Welfare Ethical Review Body (AWERB) and UK Home Office approval. C57BL/6J mice carrying the *Pax3^Cre^* knock-in allele (Lang et al., 2005) were timed-mated to mixed C57BL/6J;129/sv mice carrying a *Rosa^tdTom^* recombination reporter (Madisen et al., 2010). Cryosections of formaldehyde-fixed E12.5 liver were blocked in PBS containing 10% serum-free protein block (DAKO), 2.5% bovine serum albumin (BSA), and 0.1% Triton X-100 before staining with the following antibodies as indicated in the figures: goat anti-mouse KDR (R&D Systems, #AF644, Lot COA0420091, 1:50), rat anti-mouse CD45 (BD Biosciences, #550539, Lot 9301732, 1:50), rabbit anti-mouse RFP (MBL, #PM005, Lot 048, 1:250), rabbit anti-mouse KI67 (Abcam, #ab16667, Lot GR8416681-9, 1:100), goat anti-mouse RFP (OriGene, #AB1140-100, Lot 081290121, 1:250). This was followed by Alexa Fluor 647-conjugated donkey anti-goat Fab fragments (Stratech, #705-606-147, 1:200), Alexa Fluor 488-conjugated donkey anti-rat Fab fragments (Stratech, #712-547-003, 1:200), Cy3-conjugated donkey anti-rabbit Fab fragments (Stratech, #711-166-152, 1:200), Alexa Fluor 647-conjugated donkey anti-rabbit Fab fragments (Stratech, #711-607-003, 1:200), or Cy3-conjugated donkey anti-goat Fab fragments (Stratech, #705-167-003, 1:200). DAPI-counterstained sections were imaged on a Ti2 microscope with NIS-Elements automatic deconvolution (Nikon) and on a LSM710 confocal microscope (Zeiss).

### Flow cytometry

Embryos were collected in ice-cold FACS buffer: RPMI with 2.5% foetal bovine serum (Thermo Fisher Scientific), 100 µg/ml heparin and 50 µg/ml DNAse I (Sigma-Aldrich). Extra-embryonic and head tissues were removed for blood collection before livers were dissected and dissociated in FACS buffer with 100 µg/ml collagenase/dispase (Sigma-Aldrich) for 20 min and TrypLE (Gibco) for 3 min. Single cell suspensions from foetal liver, foetal blood or postnatal femur and tibia bone marrow, were incubated with Fc block (BioLegend) for 30 min before labelling with the following antibodies (BioLegend): KIT (clone 2B8, 1:50), CD45 (clone 30-F11, 1:50), TER119 (clone TER-119, 1:100), CD11b (clone M1/70, 1:50), CD41 (clone MWReg30, 1:50), F4/80 (clone BM8, 1:50), Lineage (Lin) cocktail (CD3ε, clone 145-2C11; Ly-6G/Ly-6C, clone RB6-8C5; CD11b, clone M1/70; CD45R/B220, clone RA3-6B2; TER-119, clone Ter-119; 1:100), CD16/32 (clone 93, 1:50), SCA1 (clone D7, 1:50). Live cells were analysed using a Fortessa X-20 (BD Biosciences) or sorted on a FACSAriaIII (BD Biosciences). Data were analysed using FlowJo VX (FlowJo) and Prism 9 (GraphPad).

### Haematopoietic differentiation assay

Haematopoietic differentiation potential was tested in CFU assays using Methocult GF-M3434 (STEMCELL Technologies), following the manufacturer's instructions.

## Supplementary Material


