# Supplementary material for: A Pax3 lineage gives rise to transient haematopoietic progenitors
Source: Development. 2024 Dec 4;151(23):dev202924. doi: 10.1242/dev.202924 (PMC11658681; doi:10.1242/dev.202924)
Supplement: Supplementary file 1 [file develop-151-202924_review_history.pdf]

A *Pax3* lineage gives rise to transient haematopoietic progenitors

Giovanni Canu, Rosamaria Correra, Guillermo Diez-Pinel, Raphaël F. P. Castellan, Laura Denti, Alessandro Fantin and Christiana Ruhrberg
DOI: 10.1242/dev.202924

Editor: Hanna Mikkola

Review timeline
Original submission: 6 April 2024
Editorial decision: 4 June 2024
First revision received: 15 October 2024
Accepted: 4 November 2024

Original submission

First decision letter

MS ID#: DEVELOP/2024/202924

MS TITLE: A Pax3 lineage gives rise to transient haematopoietic progenitors

AUTHORS: Giovanni Canu, Rosamaria Correra, Guillermo Diez-Pinel, Raphael Castellan, Laura Denti, Alessandro Fantin, and Christiana Ruhrberg

I have now received all the referees' reports on the above manuscript. The referees' comments are appended below, or you can access them online: please go to BenchPress and click on the 'Manuscripts with Decisions' queue in the Author Area.

As you will see, the referees express considerable interest in your work, but still have some significant criticisms and recommend a substantial revision of your manuscript before we can consider publication.

I would like to work with you on this, and hear from you if you can identify areas based on their recommendations that you will be able to address, vs. those that will not be feasible. Please send us a point-by-point response indicating your plans for addressing the referees' comments, and we will look over this and provide further guidance.

Please attend to all of the reviewers' comments and ensure that you clearly highlight all changes made in the revised manuscript. Please avoid using 'Tracked changes' in Word files as these are lost in PDF conversion. I should be grateful if you would also provide a point-by-point response detailing how you have dealt with the points raised by the reviewers in the 'Response to Reviewers' box. If you do not agree with any of their criticisms or suggestions please explain clearly why this is so.

Reviewer 1

Advance summary and potential significance to field

The study by the authors sheds light on the presence of a transient population of Pax3 lineage cells in the mouse fetal liver and circulation, exhibiting characteristics suggestive of hematopoietic progenitors capable of generating myeloid and erythroid cells. This observation stems from RNAseq data and subsequent validation in embryos. While the results are well-presented and convincing,

© 2024. Published by The Company of Biologists under the terms of the Creative Commons Attribution License (https://creativecommons.org/licenses/by/4.0/).

the authors stop short of providing at least some evidence regarding the specific role of this population. Furthermore, the analysis is confined to a single time point in the embryo and the genetic approach utilized (a constitutive Cre lineage tracing tg line) does not allow for pinpointing the emergence time of these cells. As such, while the observation holds potential interest, it remains speculative without further substantiation.

Comments for the author

As previously mentioned, the study's scope is limited to the observation of Pax3+ derived hematopoietic stem cells (HSCs) or potentially erythromyeloid progenitors (EMPs) at a singular time point. The absence of a comprehensive time course spanning both earlier and later developmental stages, imposes constraints on the depth of the analysis and the interpretation of the findings. Given the inherent challenges associated with isolating cells for fetal liver transplant at E12.5 exploration at later developmental time points, such as E14-E15, presents a viable avenue for extending the investigation. By conducting transplantation experiments at these later stages, the persistence, dynamics, and functional properties of the observed cell population could be more thoroughly elucidated.

<u>Reviewer 2</u>

Advance summary and potential significance to field

In the manuscript entitled “A Pax3 lineage gives rise to transient haematopoietic progenitors” by Canu et al., the authors show the presence of a transient population of Pax3 lineage hematopoietic progenitors in the fetal liver, and, to a smaller extent, in the bone marrow of P6 neonatal mice. The findings are novel and interesting and they can serve as starting point for future research on the topic.
However, it is unclear what is the role and the significance of the Pax3 lineage progenitors at this stage.

Comments for the author

After reading the rebuttal to Blood Advances' reviewers, this reviewer understands that the authors do not have sufficient resources to continue the project, but a couple of key experiments would help strengthening the findings and understanding if we are indeed talking about a subset of EMPs:

1- Are tdTom+ EMPs (defined as CD41locKit+CD16/32+) present in E12.5 FL of Pax3Cre;Rosa tdTom embryos?

2- The authors say that "these finding suggest that Pax3 lineage TER119+ cells arise locally in the fetal liver", but did they look in the yolk sac to see if EMPs (defined as CD41locKit+CD16/32+) present in the Yolk sac of Pax3Cre;Rosa tdTom embryos at E9/E9.5 are tdTom+? This might help figuring out if these progenitors are passing through the YS before seeding the fetal liver, and hence behave as YS-derived EMPs.

3- Do the tdTom+ progenitors have lymphoid potential? I understand there is no evidence of mature tdTom+ T and B cells in the FL, but what about the potential of tdTom+ progenitors? A CFU-C assay with a lymphoid-supporting methyl cellulose medium can help answering this point and clarifying if the Pax3 lineage progenitors are more like EMPs or LMPs.

4- How do tdTom+ cells differ from the tdTom- ones? This reviewer saw that this was previously asked by Blood Avdances' reviewers, but still thinks this is an important point to address. Can the authors repeat the experiments shown in the response to Reviewer #1 (see "Response to reviewers from Blood Advances" document) to be sure there are no differences at least in CFU-C assay and include the data in the manuscript?

Minor points:
1- Do the authors know which kind of cells are tdTom high in the FL?

© 2024. Published by The Company of Biologists under the terms of the Creative Commons Attribution License (https://creativecommons.org/licenses/by/4.0/).

2- In the original publication "Singh AJ, Chang CN, Ma HY, Ramsey SA, Filtz TM, Kioussi C. FACS-Seq analysis of Pax3-derived cells identifies non-myogenic lineages in the embryonic forelimb. Sci Reports 2018 81.
2018;8(1):1-16. doi:10.1038/s41598-018-25998-1" the mouse model used is Pax3Cre;RosaEGFP and not RosaYfp. The text and the figures should be modified accordingly.

Reviewer 3

Advance summary and potential significance to field

This is an interesting study of the contribution of Pax3+ cells to endothelial and hematopoietic progenitor cell populations in the fetal liver. The information provided is novel and should be of interest to the field, but there are some questions that could be addressed to make the story stronger.

Comments for the author

1. The presence of Pax3-lineage (tdTom+) cells in the fetal liver and blood is convincing, but what is the origin of these endothelial and hematopoietic cells therein? It is suggested that they arise within the fetal liver, but are these cells present in the yolk sac or AGM region, as well as blood, at earlier stages of development (E9.5-11.5)? It is possible that circulating cells from these other tissues accumulate (and possibly propagate) in the fetal liver as transient populations, especially since they are detected in circulation, and this would be interesting/important to know. This investigation is suggested as a future direction, but the results would add a lot to this study.
2. A related question - what is the expression pattern of endogenous Pax3 in early and mid-gestation hematopoietic sites?
3. What is the half-life of tdTom signal in this animal model?
4. Are the tdTom+CD45+ cells in the fetal liver actively proliferating (for example, pHH3+ or EdU+), as other hematopoietic progenitors do?
5. Do the tdTom+KDR+ “endothelial cells” in the fetal liver tissues express other markers of this cell type, such as VE-Cadherin, eNOS, etc?
6. In the KIT+Sca+Lin- (KSL, hematopoietic stem/progenitor cells) population in fetal liver and blood over the developmental time course, what proportion of that is tdTom+?
7. Similarly, what is the proportion in postnatal bone marrow KSL, related to Figure 3C?
8. The last 2 paragraphs of the Results sections would be more appropriate in the Discussion.

First revision

Author response to reviewers' comments

Response to Reviewer #1
***** Reviewer 1 Advance summary and potential significance to field
The study by the authors sheds light on the presence of a transient population of Pax3 lineage cells in the mouse fetal liver and circulation, exhibiting characteristics suggestive of hematopoietic progenitors capable of generating myeloid and erythroid cells. This observation stems from RNAseq data and subsequent validation in embryos. While the results are well-presented and convincing, the authors stop short of providing at least some evidence regarding the specific role of this population. Furthermore, the analysis is confined to a single time point in the embryo and the genetic approach utilized (a constitutive Cre lineage tracing tg line) does not allow for pinpointing the emergence time of these cells. As such, while the observation holds potential interest, it remains speculative without further substantiation.
Reviewer 1 Comments for the author
As previously mentioned, the study's scope is limited to the observation of Pax3+ derived hematopoietic stem cells (HSCs) or potentially erythromyeloid progenitors (EMPs) at a singular time point. The absence of a comprehensive time course, spanning both earlier and later developmental stages, imposes constraints on the depth of the analysis and the interpretation of the findings.

© 2024. Published by The Company of Biologists under the terms of the Creative Commons Attribution License (https://creativecommons.org/licenses/by/4.0/).

Reply: Our analysis is not confined to a single time point. In Fig. 2A,B, we show a time course for the number of Pax3 lineage cells in both liver and blood ranging from E11.5 to E14.5. In this time window, we focused our analyses on the E12.5 time point, when we detected the highest number of Pax3 lineage haematopoietic cells. We also examined postnatal development at P6 and P10 (previously Fig. 3C, now Fig. 3F), and we have now added new data to examine whether the Pax3 lineage resemble EMPs or LSK cells at E12.5 or E15.5 (new Fig. 3C,E).

Reviewer 1: Given the inherent challenges associated with isolating cells for fetal liver transplant at E12.5, exploration at later developmental time points, such as E14-E15, presents a viable avenue for extending the investigation. By conducting transplantation experiments at these later stages, the persistence, dynamics, and functional properties of the observed cell population could be more thoroughly elucidated.
Reply: In our opinion, this experiment is unlikely to be informative, because the Pax3 lineage-traced progenitors are more similar to EMPs than HSCs, as discussed in the manuscript (lines 128-130 and 141-150). For example, Pax3 lineage-traced progenitors accumulate in the foetal liver before HSCs but rapidly decline after E12.5 when HSC numbers in the liver rise, and unlike HSCs, they are also absent from the post-natal bone marrow. Therefore, a transplantation experiment would not likely result in engraftment. Given the large number of mice required to conclusively demonstrate a likely negative result, we also do not consider this experiment ethically justifiable. Thus, isolating the appropriate number of E14.5 Pax3 lineage-traced progenitors for transplantation experiments would require an estimated 50 embryos as donors to obtain at least 10,000 Pax3 lineage KIT+ CD45+ cells, a typical number of cells used for transplantation; assuming an average litter size of 8 and an expected Mendelian inheritance of 50% for the lineage trace, we would need to breed and cull at least 12 females for a single transplantation experiment, which then would have to be repeated multiple times and may still not be conclusive, considering the similarity of the Pax3 lineage KIT+ CD45+ cells to EMPs and not HSCs.

***** Reviewer 2 Advance summary and potential significance to field
In the manuscript entitled “A Pax3 lineage gives rise to transient haematopoietic progenitors” by Canu et al., the authors show the presence of a transient population of Pax3 lineage hematopoietic progenitors in the fetal liver, and, to a smaller extent, in the bone marrow of P6 neonatal mice. The findings are novel and interesting and they can serve as starting point for future research on the topic. However, it is unclear what is the role and the significance of the Pax3 lineage progenitors at this stage.

Reviewer 2 Comments for the author
After reading the rebuttal to Blood Advances' reviewers, this reviewer understands that the authors do not have sufficient resources to continue the project, but a couple of key experiments would help strengthening the findings and understanding if we are indeed talking about a subset of EMPs:

1- Are tdTom+ EMPs (defined as CD41lo cKit+ CD16/32+) present in E12.5 FL of Pax3Cre;Rosa tdTom embryos?
Reply: We have now performed this experiment and found that Pax3 lineage EMPs are present in the E12.5 FL, but decline by E15.5 (new Fig. 3C).

2- The authors say that "these finding suggest that Pax3 lineage TER119+ cells arise locally in the fetal liver", but did they look in the yolk sac to see if EMPs (defined as CD41locKit+CD16/32+) present in the Yolk sac of Pax3Cre;Rosa tdTom embryos at E9/E9.5 are tdTom+? This might help figuring out if these progenitors are passing through the YS before seeding the fetal liver, and hence behave as YS-derived EMPs.

Reply: Immunostaining of the E8.5 yolk sac did not identify Pax3 lineage cells (new Fig. 3D). Furthermore, bioinformatic analysis of published single-cell RNA sequencing (scRNAseq) data from mouse embryo and yolk sac (PMID: 37590359) showed that Pax3 gene expression was confined to paraxial mesoderm and neural crest derivatives but absent from yolk sac endothelium or haematopoietic cells at E8.5 (Reviewer Figure, supplemental).

NOTE: We have removed unpublished data that had been provided for the referees in confidence.

© 2024. Published by The Company of Biologists under the terms of the Creative Commons Attribution License (https://creativecommons.org/licenses/by/4.0/).

3- Do the tdTom+ progenitors have lymphoid potential? I understand there is no evidence of mature tdTom+ T and B cells in the FL, but what about the potential of tdTom+ progenitors? A CFU-C assay with a lymphoid-supporting methyl cellulose medium can help answering this point and clarifying if the Pax3 lineage progenitors are more like EMPs or LMPs.
Reply: To the best of our knowledge, CFU assays do not allow monitoring lymphocyte potential, which instead is usually induced in suspension cultures using lymphocyte-inducing media and co-culture with OP9 stromal cells overexpressing DLL4. This method requires extended culture times (usually 4-5 weeks), after which lymphocyte differentiation is assessed by flow cytometry monitoring the expression of lymphocyte markers such as CD3/CD4/CD8. Notably, such differentiation cultures have limited efficiency even with proliferating cord blood HSCs, and thus would require many embryos to obtain sufficient cells to reach a conclusive answer. We do not have experience setting up these complex culture conditions. In any case, culture experiments would merely test potential, not in vivo significance. In this context, we should consider that only few lymphocytes arise at E12.5, and that our RNAseq analysis did not identify differentiated lymphocyte markers in the Pax3 lineage (Fig. 1A), Further, our new data (Fig. 3C) reinforce the idea that the Pax3 lineage progenitor cells resemble EMPs with predominant erythroid and myeloid potential. In view of these considerations, we hope the reviewer agrees that testing for lymphocyte potential is not a current priority.

4- How do tdTom+ cells differ from the tdTom- ones? This reviewer saw that this was previously asked by Blood Avdances' reviewers, but still thinks this is an important point to address. Can the authors repeat the experiments shown in the response to Reviewer #1 (see "Response to reviewers from Blood Advances" document) to be sure there are no differences at least in CFU-C assay and include the data in the manuscript?
Reply: We have now repeated the CFU experiment, which confirmed that there is no significant difference in the differentiation potential of tdTOM- and tdTOM+ progenitors (new Fig. 4D).

Minor points:
1- Do the authors know which kind of cells are tdTom high in the FL?
Reply: Fig. 1C shows that a small fraction of Pax3 lineage cells is tdTom high in the FL, and that these cells are negative for both KDR or CD45, excluding haematopoietic and endothelial identity. We have no further knowledge beyond these characteristics, and therefore cannot comment on the identity of these cells.

2- In the original publication "Singh AJ, Chang CN, Ma HY, Ramsey SA, Filtz TM, Kioussi C. FACS-Seq analysis of Pax3-derived cells identifies non-myogenic lineages in the embryonic forelimb. Sci Reports 2018 81. 2018;8(1):1-16. doi:10.1038/s41598-018-25998-1" the mouse model used is Pax3Cre;RosaEGFP and not RosaYfp. The text and the figures should be modified accordingly.
Reply: We have corrected the manuscript as advised.

***** Reviewer 3 Advance summary and potential significance to field
This is an interesting study of the contribution of Pax3+ cells to endothelial and hematopoietic progenitor cell populations in the fetal liver. The information provided is novel and should be of interest to the field, but there are some questions that could be addressed to make the story stronger.

Reviewer 3 Comments for the author
1. The presence of Pax3-lineage (tdTom+) cells in the fetal liver and blood is convincing, but what is the origin of these endothelial and hematopoietic cells therein? It is suggested that they arise within the fetal liver, but are these cells present in the yolk sac or AGM region, as well as blood, at earlier stages of development (E9.5-11.5)? It is possible that circulating cells from these other tissues accumulate (and possibly propagate) in the fetal liver as transient populations, especially since they are detected in circulation, and this would be interesting/important to know. This investigation is suggested as a future direction, but the results would add a lot to this study.
Reply: Apologies for giving the impression that we consider the progenitors to be originating in the foetal liver. We only know that they accumulate in the foetal liver at E12.5, suggesting that they are homing to the foetal liver, similar to other foetal haematopoietic progenitors. We have now clarified this in the manuscript (lines 106-112). Notably, very few Pax3 lineage cells can be detected in the foetal liver or circulation at E11.5 (1.8%, Fig. 2A,B), and very few or no tdTOM+

© 2024. Published by The Company of Biologists under the terms of the Creative Commons Attribution License (https://creativecommons.org/licenses/by/4.0/).

cells were present at E10.5 (<1%). We assessed E8.5 yolk sacs by immunostaining but did not identify any Pax3 lineage cells in this tissue (new Fig. 3D). Furthermore, bioinformatic analysis of published scRNAseq datasets (PMID: 37590359) from mouse embryo and yolk sac at E8.5 showed Pax3 gene expression in paraxial mesoderm and neural crest derivatives but not yolk sac endothelial and haematopoietic cells (Reviewer Fig. R3). We conclude that Pax3 progenitors are either generated from a haemogenic source inside the embryo, or that they express Pax3 only after leaving the yolk sac. It is conceivable that the novel haematopoietic progenitors identified here might themselves lack Pax3 expression, but arise from Pax3-expressing cells, with candidates being Pax3-derived endothelium with haemogenic properties, perhaps in the paraxial mesoderm, neural tube or foetal liver. To address all these possibilities comprises a substantial amount of additional work. As we do not have funding to carry out further haematopoietic work, we have chosen the format of a short report to disclose our conclusive findings to date whilst opening doors to others potentially interested in this novel lineage and their origins.

2. A related question - what is the expression pattern of endogenous Pax3 in early and mid-gestation hematopoietic sites?
Reply: Pax3 is considered to be a specific marker of paraxial mesoderm and neural crest and has not previously been reported to be expressed in any early or mid-gestation haematopoietic sites. Our analysis of published scRNAseq data (PMID: 37590359) shows that Pax3 transcripts are not detected in the yolk sac or in haematopoietic cells at E8.5 (Reviewer Fig. 3). However, we must consider that Pax3 is a relatively lowly expressed gene and may therefore remain undetected by scRNAseq, which is the reason we decided against including these scRNAseq data in the current manuscript. Nevertheless, Pax3 lineage-tracing also did not identify Pax3 lineage cells in the yolk sac, even though we could identify lineage tracing of pre-somitic and neural tube cells in the embryo proper (new Fig. 3D). Detection of potential Pax3 lineage haemogenic cells in the AGM by immunostaining would be more challenging, because this region is surrounded by somitic mesoderm, which expresses Pax3 at very high levels. Further, there is presently no evidence that the Pax3 lineage progenitors resemble HSCs, which arise from the AGM, and they instead appear more similar to EMPs.

3. What is the half-life of tdTom signal in this animal model?
Reply: The genetic system used for our study relies on the irreversible recombination of the tdTom reporter after the onset of Cre expression. As a result, each cell that derives from a Pax3-expressing progenitor will continuously and permanently express tdTOM even when Pax3 is no longer expressed.

Reviewer 3:
4. Are the tdTom+CD45+ cells in the fetal liver actively proliferating (for example, pHH3+ or EdU+), as other hematopoietic progenitors do?
Reply: We have immunostained foetal liver sections to include the proliferation marker KI67. These experiments show that tdTOM+CD45+ cells indeed are actively proliferating (new Fig. 4A). The ability to proliferate is also confirmed by the capacity of these cells, once sorted, to proliferate and differentiate in vitro in a CFU assay (Fig. 4B-D).

5. Do the tdTom+KDR+ “endothelial cells” in the fetal liver tissues express other markers of this cell type, such as VE-Cadherin, eNOS, etc?
Reply: We note that Pax3 lineage endothelial cells have been described by others, including in the foetal liver (e.g., PMID: 31130354), and we included EC data simply to distinguish endothelial cells from the novel Pax3 lineage haematopoietic cells.

6. In the KIT+Sca+Lin- (KSL, hematopoietic stem/progenitor cells) population in fetal liver and blood over the developmental time course, what proportion of that is tdTom+?
Reply: We have repeated our experiments and found that the Pax3 lineage includes LSK cells in the E12.5 foetal liver, and that their number declines by E15.5 (new Fig. 3E). These markers are commonly used to define a haematopoietic stem/progenitor pool in the foetal liver. However, their rapid decline thereafter, and the lack of Pax3 lineage haematopoietic progenitors in the postnatal bone marrow (new Fig. 3F) suggest that the Pax3 lineage population does not contain HSCs, but rather EMP-like progenitors.

7. Similarly, what is the proportion in postnatal bone marrow KSL, related to Figure 3C?

© 2024. Published by The Company of Biologists under the terms of the Creative Commons Attribution License (https://creativecommons.org/licenses/by/4.0/).

Reply: The figure referred by the Reviewer (now Fig. 3F) shows complete absence of KIT+ cells in the residual Pax3 lineage population present in the bone marrow, so we could not investigate the LSK lineage at this stage.

8. The last 2 paragraphs of the Results sections would be more appropriate in the Discussion.
Reply: The paragraphs are currently included in a combined Results and Discussion section, due to the format of a short report for this journal.

Second decision letter

MS ID#: DEVELOP/2024/202924

MS TITLE: A Pax3 lineage gives rise to transient haematopoietic progenitors

AUTHORS: Giovanni Canu, Rosamaria Correra, Guillermo Diez-Pinel, Raphael Castellan, Laura Denti, Alessandro Fantin, and Christiana Ruhrberg

I am happy to tell you that your manuscript has been accepted for publication in Development, pending our standard publication integrity checks.

Reviewer 2

Advance summary and potential significance to field

In the manuscript entitled "A Pax3 lineage gives rise to transient haematopoietic progenitors" by Canu et al., the authors show the presence of a transient population of Pax3 lineage hematopoietic progenitors in the fetal liver, and, to a smaller extent, in the bone marrow of P6 neonatal mice. The findings are novel and interesting and they can serve as starting point for future research on the topic.

Comments for the author

The revised version of the manuscript shows improvement compared to the first submission. While the relevance of the novel, and interesting, subset of Pax3-derived EMP-like progenitors is still not clear, and the findings remain descriptive, this reviewer thinks that the format of the short report chosen by the authors is a good fit for publishing this work. This reviewer is satisfied with the answers given by the authors and thinks the work is suitable for publication in its current form.

Reviewer 3

Advance summary and potential significance to field

Previously provided.

Comments for the author

This revised manuscript adequately addresses previous concerns, and will be of interest to the field.

© 2024. Published by The Company of Biologists under the terms of the Creative Commons Attribution License (https://creativecommons.org/licenses/by/4.0/).
